# Social Motivation to Comply with COVID-19 Guidelines in Daily Life in South Korea and the United States

**DOI:** 10.3390/bs12070213

**Published:** 2022-06-27

**Authors:** Min Young Kim, Kyueun Han

**Affiliations:** 1 Department of Psychology, Keimyung University, Daegu 42602, Korea; mkim@kmu.ac.kr; 2 College of Kyedang General Education, Sangmyung University, Seoul 03016, Korea

**Keywords:** collectivism, compliance with COVID-19 guidelines, impression management

## Abstract

Collectivism assessed at the national level has been suggested as a psychological factor that affects compliance with COVID-19 guidelines in daily life. The level of assessment and conceptual construct of collectivism, however, vary across studies, which calls for the need to clarify the power of collectivism in explaining individuals’ compliance behaviour. With this aim, we investigated individual-level collectivism, the unique variance and other relevant factors, such as altruism (e.g., for the family, community, and humanity) and impression management (e.g., what others would think of me) in explaining compliance with COVID-19 guidelines in US and South Korean participants. The results of hierarchical regression analysis showed that collectivism was a significant factor that explained compliance only in the US participants, whereas impression management was significant and explained the additional variance over collectivism in compliance in both the US and South Korean participants. The findings suggest the importance of elucidating the overlap between collectivism and impression management in studies exploring COVID-19 guideline adherence in daily life.

## 1. Introduction

Owing to the COVID-19 pandemic, strict government interventions have been implemented worldwide for more than two years, as of 2022. The analysis of location data from billions of Google users’ phones showed that individuals in Eastern countries, such as South Korea, were more cooperative than those in Western countries, such as the United States (US) and the United Kingdom (UK) [1]. For example, wearing facial masks to prevent the spread of the virus is a controversial issue in Western countries. A congress member who refused to wear a mask in the US was fined approximately 48,000 dollars for violating the COVID-19 guidelines [2]. Additionally, protests rose in Calgary, Canada, demanding freedom to remove masks [3]. Notably, such movements did not occur in Eastern countries. As South Koreans have a more salient social structure and interpersonal relationships based on the concept of ‘we’, which refers to we-ness, they comply more with mask-wearing behaviour [4]. The question is, what kind of cultural differences affects attitudes and behaviours towards compliance with COVID-19 guidelines?

Since the World Health Organization (WHO) declared COVID-19 a pandemic on 11 March 2020, researchers have tried to identify psychological factors that predict compliance with government interventions in order to curb the spread of the virus. Regarding social organisation, most studies have suggested that individualism or collectivism is a critical factor that causes cultural differences in behaviour [5,6]. In a province-level study in China, Huang et al. showed that higher collectivism was associated with a stronger tendency for people to comply with COVID-19 guidelines [7]. At the national level, high collectivism has also been found to be associated with fewer confirmed cases and lower mortality in 69 countries [8]. Similarly, in a study of 54 countries, those with collectivist cultures showed a lower rate of increase in COVID-19 cases than those with individualistic cultures [9].

### 1.1. Individual-Level Collectivism

Based on pioneering studies, more specific approaches are required to understand cultural differences. One such approach is to assess collectivism at an individual level. As most of the analyses in the literature were conducted using public data from different countries, and not individual data, it was impossible to understand the contributing psychological factors at the individual level. In other words, if the unit of analysis is a country, the relationship can be inferred from existing national data on collectivism, and overall group differences can be found across countries, but the psychological dynamics occurring within an individual remain undiscovered. Although a few studies have attempted individual-level analyses, it is difficult to generalise the results because the analyses were conducted mostly in countries with individualistic cultures, such as the US and the UK [10]. Hence, further research is needed to investigate psychological dynamics at the individual level in countries with collectivist cultures.

**Figure 1:**
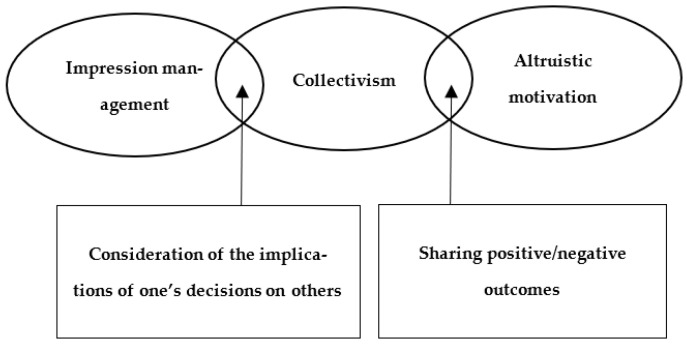
A conceptual model of this study.

The concept of collectivism is multidimensional and includes various sub-concepts [5,11]. As described by Brewer and Chen, the way collectivism has been assessed and defined in literature is ‘overly broad and diffuse’ [12] (p. 134). Researchers have also suggested that the operationalisation of collectivism is highly varied, such that it seems to be a catch-all concept for various forms of cultural differences [12,13]. In this sense, it has been suggested that the definition of collectivism may even include unrelated constructs such as communitarianism or altruism that do not necessarily vary in terms of collectivism [13]. The potential conceptual convergence between collectivism and other related constructs requires revisiting the previous findings on the association between collectivism and compliance with group rules [12]. Based on the proposal of Cross et al. [14], suggesting that the core aspects of collectivism are being considerate of the implications of one’s decisions on others and sharing of positive and negative outcomes, we developed a conceptual model in which factors of collectivism may overlap with impression management (IM) or altruism (Figure 1).

### 1.2. Social Motives

First, the aspect of collectivism that describes the consideration of implications of one’s decisions on others can be similar to some aspects of IM. IM is driven by a social motive to control information to influence the impressions formed by others [15]. As people who consider the implications of their decisions on others place a greater emphasis on interpersonal relationships, fitting into a society by making a socially desirable decision is valued more in collectivist than in individualistic cultures [16,17]. Thus, people in collectivist cultures are more likely to behave in a socially oriented manner to demonstrate that they care about social norms in an overt way [18]. Given the conceptual overlap between collectivism and IM, the observed compliance with COVID-19 guidelines might not only be due to collectivist motives but also due to IM motives (Figure 1). For example, individuals may sacrifice their freedom and wear masks during the COVID-19 pandemic partially to be perceived as socially acceptable by others.

Research on socially desirable responses supports the idea that collectivism and IM share some common variance. A study has shown that the motivation to fit in within a collectivist society triggers the desire in individuals for IM by being normatively appropriate [19]. Collectivist cultures have more pervasive social norms and less tolerance for deviance than individualistic ones; this cultural demand leads to strong disapproval of anyone defying their duties and obligations as a group member [9]. Hence, showing normative responses helps people meet the cultural demand and maintain a desired social image or identity, particularly in collectivist cultures. Managing one’s impression in line with normative responses is also relatively easy in collectivist cultures [18].

The findings from previous studies suggest the possibility of a conceptual overlap between collectivism and altruism. An additional aspect of collectivism, sharing positive/negative outcomes with relevant others, may have similarities with altruism. Altruism refers to the willingness to make major personal sacrifices to aid people for the benefit of others [20]. As people should aim to achieve the interests and goals of the group in collectivist culture, collectivism and altruism are similar because people with both motives sometimes disregard their personal goals for the sake of others (i.e., altruism) or to achieve communal goals for the groups (i.e., collectivism) [21,22]. Based on the convergence of collectivism and altruism, a measure of collectivism directly assesses the degree to which individuals are willing to sacrifice their personal interests for the benefit of the group when the two are incompatible [23]. With the conceptual overlap between collectivism and altruism, observed compliance with COVID-19 guidelines could occur because of collectivist as well as altruistic motives (Figure 1). For instance, some individuals may sacrifice their freedom and wear masks partially out of concern for the well-being of others.

Empirical studies support this idea by showing that collectivism is positively correlated with altruism. People with higher collectivism have reported higher levels of altruism [24,25]. According to a recent study, altruistic motives predict the degree to which people comply with COVID-19 hygiene regulations [26]. Given that collectivism and altruism are overlapping constructs, it is necessary to investigate the relative explanatory power of collectivism by considering altruism.

### 1.3. Present Study

This study was designed to expand on preliminary studies on collectivism and behaviour during the COVID-19 pandemic. For this, we assessed collectivism at the individual level and examined whether it predicted compliance with COVID-19 guidelines in an individualistic (the US) and a collectivist (South Korea) culture group. Furthermore, we included variables such as altruistic motives and IM to determine the unique variance contributed by collectivism. While assessing motivation, we asked participants about situation-specific motivation directly relevant to COVID-19. In particular, domain-general motivations refer to internalised motivations that emerge across individuals’ cumulative history of life experiences, while situation-specific motivations refer to motivations that emerge in immediate response in a particular context [27]. As situation-specific constructs can explain behaviours in the specific context better than the domain-general components [28], we specified motivations of altruism and IM for compliance with COVID-19 guidelines for a better explanation of behaviours during the pandemic. Thus, the aim of this study was to explore the relative importance of collectivism and social motives that contribute to compliance with COVID-19 guidelines. In the following section, we present the measures and analyses of this study, followed by the results. In the discussion, we outline the implications and limitations of the study.

**Hypothesis** **1.** *IM motives explain theunique variance of compliance behaviour over collectivism*.

**Hypothesis** **2.** *Altruistic motives explain the unique variance of compliance behaviour over collectivism*.

**Hypothesis** **3.** *The relative importance of collectivism differs across cultures*.

## 2. Materials and Methods

### 2.1. Measurement

*Collectivism*. The self-construal scale developed by Singelis was used to assess the collectivist characteristics of participants [17]. The scale showed convergent and divergent validity in measuring collectivism in both the US and Korea [29]. Based on a recent study on its factor structure, 11 items for the Korean participants and 12 items for the US participants were averaged and used as the collectivism scores, respectively [30]. Participants were asked to rate the degree to which they agreed with each statement using a seven-point Likert scale ranging from 1 (*strongly disagree*) to 7 (*strongly agree*). A sample item is, ‘My happiness depends on the happiness of those around me’.

*Altruistic and IM Motives*. Participants were asked whether they complied with the COVID-19 guidelines for personal protection (‘for one’s own health’), altruistic motives (‘for the family’, ‘for the community’, ‘for the safety of mankind’), and IM motives (‘because of what others think’). English versions were translated into Korean and verified by bilingual users.

*Social Distancing.* Participants reported their degree of compliance with COVID-19 guidelines (e.g., crowd avoidance, distance maintenance, and hand washing) on an 11-point scale ranging from 0 (*never*) to 10 (*maintain all the time*).

*Demographics*. Demographic information such as age, sex, country of birth, and residence were assessed.

### 2.2. Participants

Prior to the study, we performed an a priori statistical power analysis for sample size estimation in G*Power using a linear multiple regression: Fixed model, R^2^ increase [31]. A power of 0.95, an α level of 0.05, and a medium effect size (f^2^ = 0.15) require a sample size of approximately at least 107 participants in each sample. A total of 210 US-based participants (M_age_ = 36.47, SD_age_ = 10.68, 34.8%) were recruited via Amazon’s Mechanical Turk (www.MTurk.com) accessed on 18–22 August 2020. The inclusion criteria were as follows: participants who were born or have lived in the US for at least 20 years (M_year_ = 33.75, SD_year_ = 11.95). All participants received a cash reward ($1.00) to complete the survey. In addition, 197 participants (M_age_ = 23.71, SD_age_ = 7.14, 69.0%) were recruited from South Korea. Korean participants were given course credits or a reward of KRW 1000.

### 2.3. Procedures

The survey was conducted online. The survey was conducted online via Amazon’s Mechanical Turk (www.Mturk.com) accessed on 18–22 August 2020. Participants voluntarily clicked a link in the survey list and completed the study. Participants were told that it was a study on the outbreak of the COVID-19 pandemic and consequent changes in daily life. Those who volunteered to participate agreed to the online consent form and completed the survey, which included items on collectivism, social motivation (altruistic and IM motives), social distancing behaviour, and demographics. Different versions of the survey were created in the respondents’ native language (English and Korean). All items were translated by bilingual users and other bilingual users reviewed and approved the translation through forward and reverse translation. The Ethics Committee for Human Research of the relevant institution approved this study protocol.

## 3. Results

Table 1 displays the descriptive statistics of the study and correlations among the variables. The results are summarised according to cultural groups. We conducted two separate hierarchical regression analyses with compliance behaviour as a dependent variable for the US and Korean participants to test the additional variance explained by altruistic and IM motives over collectivism. In each model, age, gender, and level of collectivism were entered in Step 1, and altruistic motivation and IM motivation were entered in Step 2.

The results of the hierarchical regression analysis showed that in Step 1 for the US sample, when the independent variables of age, gender, and collectivism were entered, 12.3% of the total variance was explained (R^2^ = 0.123, F (3, 206) = 9.600, *p* < 0.000). In Step 2, when IM motive, altruistic motives (i.e., for community and humanity), and motivation for one’s own health were entered, an additional 10.6% of the variance was explained on top of Step 1 (R^2^ change = 0.106, F_change_ (4, 202) = 9.906, *p* < 0.000). The altruistic motive, ‘for my family’ was excluded from the final model. Specifically, both collectivism (b = 0.366, *p* < 0.001) and IM motive (b = −0.284, *p* < 0.001) significantly explained participants’ compliance with the COVID-19 guidelines in the US sample (Table 2).

In the Korean sample, in Step 1, when independent variables of age, gender, and collectivism were entered, 5.4% of the total variance was explained (R^2^ = 0.054, F (3, 191) = 3.613, *p* = 0.014). In Step 2, when IM motive, altruistic motives (i.e., for my family, community, and humanity) were entered, an additional 8.3% of variance was explained on top of Step 1 (R^2^_change_ = 0.083, F_change_ (4, 187) = 4.466, *p* = 0.002). Non-altruistic motives (i.e., for one’s own health) were excluded from the final model. Specifically, age (b = 0.183, *p* = 0.024) and IM motive (b = −0.300, *p* < 0.001) significantly explained participants’ compliance with the COVID-19 guidelines in the Korean sample (Table 2).

Overall, IM motive explained the compliance behaviour in both samples, which supports Hypothesis 1. Altruistic motive explained the unique variance of compliance behaviour over collectivism in the Korean sample only, which partially supports hypothesis 2. In addition, differences across samples were found in the amount of variance explained by collectivism, which supports Hypothesis 3.

## 4. Discussion

The COVID-19 pandemic requires large-scale behavioural changes which necessitate advancing our understanding of social and psychological determinants of health behaviours in addition to biomedical solutions. One such behaviour is compliance with COVID-19 guidelines, such as wearing a mask. Along with previous findings on compliance with government interventions against COVID-19, this study investigated whether collectivism at an individual level predicts compliance behaviour. In addition, as collectivism includes shared constructs with social motives, such as IM and altruism, we hypothesized that such social motives could uniquely or complementarily explain compliance with COVID-19 guidelines in daily life. The findings of this study suggest that collectivism might not be cross-culturally relevant in explaining compliance. Consistent with previous research [10,32], collectivism had significant predictive power in explaining the compliance of US participants; however, this was not observed for Korean participants. This could be explained by the fact that existing studies have mostly considered participants from Western cultures [8]. In Korean culture, where collectivism permeates everyday life (i.e., domain-general motive), the variance of collectivism across individuals may have less influence on explaining the variance of behaviour.

The findings suggest that collectivism may not be the only factor in explaining compliance with COVID-19 guidelines. Consistent with the criticism of the umbrella concept of collectivism, which covers all forms of cultural differences [12], this study suggests that collectivism may not be a dominant construct to understand compliance with COVID-19 guidelines across cultures. The previously found significance of collectivism might be the *overlap* between collectivism and other relevant concepts such as IM. Furthermore, because IM explained variance in behaviour over and above collectivism, it is possible that factors other than collectivism were excluded from previous investigations. Thus, our finding calls for the development of a more refined approach to capture each unique dimension of collectivism and other relevant factors separately and inclusively.

From a theoretical perspective, the significance of IM motives along with collectivism can be found in the social nature of the self. Managing one’s impression is essential because people validate their self-perception through the consensus of people around them [33]. Cooley, a sociologist, also stated that the ability of individuals to imagine how others evaluate them contributed to forming an image of the self [34]. In other words, an individual’s self-perception of how others perceive them is a crucial part of self-identity. Such a self-perception could lead people to engage in conformist conduct [35]. For this reason, regardless of culture, people may comply with COVID-19 guidelines to create a self-image of a collectivist or prosocial person. That is, behaving in socially desirable ways in a crisis could be non-culturally specific [36], although previous research on IM suggests that culture can mould the way people present themselves [37,38]. In this sense, we believe that identifying these kinds of psychological variables may have implications for developing policies that promote public compliance with social norms such as COVID-19 guidelines [39]. In the long run, such an approach would enrich our understanding of the role of social motives during a pandemic or crises in the future.

The limitations of this study include the representativeness of the sample. As this is a preliminary study, participants from two countries representing Western and Eastern cultures were included. In future, it will be necessary to recruit participants from various countries to generalise the results. In addition, although gender and age did not show significant explanatory power in this study, it would be necessary to employ even gender ratios and similar age ranges within the samples to enhance the quality of the results. Identifying characteristics of the sample in terms of personal experience with COVID-19, such as whether participants had overcome COVID-19 or whether they were vaccinated or not, would also help to strengthen the findings of this study. Another issue is that the findings are limited to the compliance of COVID-19 guidelines. In this study, situation-specific motives and behaviours relevant to COVID-19 were examined. Because behaviour under situational motives is likely to differ from domain-general behaviours, temperamental characteristics, general behaviour, or general cultural differences should not be inferred based on the findings of the study.

## 5. Conclusions

Using a psychological approach, this study investigated underlying social compliance behaviour across cultures during the COVID-19 pandemic. While collectivism has been suggested as a single prominent factor that explains the compliance behaviour, it may not be the case in collectivistic cultures such as Korea. The study findings suggest that social motives such as that of IM might ultimately be used to induce compliance with regulations to combat the COVID-19 pandemic. We believe that the implications of our findings are not necessarily limited to the pandemic; they can also be applied to understand the behaviour of individuals under any national-level crises that might occur in the future. Further research exploring the psychological mechanism of social motives in affecting behaviour is warranted.

## Figures and Tables

**Table 1:** Descriptive statistics and correlations among variables.

	M	SD	1	2	3	4	5	6	7	8
(a) The US										
1. Age	36.47	10.68								
2. Gender	-	-	0.082							
3. Compliance with COVID-19 guidelines	7.45	2.10	0.094	.091						
4. Collectivism	5.00	0.90	−0.020	0.082	0.331 **					
5. IM motive	15.75	9.87	−0.172 *	−0.020	−0.264 **	0.119				
6. Non-altruistic motive: For my health	23.22	14.72	0.119	−0.094	0.137 *	−0.096	−0.336 **			
7. Altruistic motive: For the health of my family	23.79	15.07	0.054	0.095	0.159 *	−0.032	−0.454 **	−0.117		
8. Altruistic motive: For the safety of my community	19.25	11.53	−0.053	−0.021	−0.005	0.042	0.131	−0.436 **	−0.334 **	
9. Altruistic motive: For humanity	17.99	12.44	−0.022	0.032	−0.140 *	0.019	0.033	−0.370 **	−0.402 **	−0.109
(b) South Korea										
1. Age	23.71	7.14	-							
2. Gender	-	-	−0.001							
3. Compliance with COVID-19 guidelines	7.43	1.91	0.146 *	0.123						
4. Collectivism	0.69	0.90	0.510 **	0.158 *	.035					
5. IM motive	0.11	1.87	0.132	0.135 *	−0.286 **	.091				
6. Non-altruistic motive: For my health	4.07	19.59	−0.127	−0.032	−0.110	.000	−0.211 **			
7. Altruistic motive: For the health of my family	35.02	19.89	−0.011	0.022	−0.058	−0.040	−0.177 **	−0.311 **		
8. Altruistic motive: For the safety of my community	14.40	14.62	0.027	0.085	0.065	0.114	−0.103	−0.365 **	−0.349 **	
9. Altruistic motive: For humanity	9.39	13.86	0.235 **	0.031	0.081	−0.142 *	−0.129	−0.351 **	−0.437 **	0.064

Note: ‘Altruistic motive: For the health of my family’ was excluded; ‘Non-altruistic motive: For my health’ was excluded; ** *p* < 0.01.; * *p* < 0.05.

**Table 2:** Regression analysis summary for each group.

	Step 1			Step 2		
	Beta	t	Sig.	Beta	T	Sig.
(a) The US						
Age	0.096	0.462	0.145	0.041	0.648	0.518
Gender	0.056	0.848	0.397	0.060	0.954	0.341
Collectivism	0.328	5.011	0.000	0.366	5.851	0.000
IM motive				−0.284	−4.236	0.000
Non-altruistic motive: For my health				0.044	0.532	0.595
Altruistic motive: For the safety of my community				0.027	0.366	0.715
Altruistic motive: For humanity				−0.119	−1.686	0.093
(b) South Korea						
Age	0.210	0.543	0.012	0.183	2.270	0.024
Gender	0.168	0.343	0.020	0.126	1.778	0.077
Collectivism	0.098	0.174	0.242	0.101	1.235	0.218
IM motive				−0.300	−4.079	0.000
Altruistic motive: For the health of my family				−0.135	−1.605	0.110
Altruistic motive: For the safety of my community				−0.036	−0.474	0.636
Altruistic motive: For humanity				−0.018	−0.228	0.820

## Data Availability

The data presented in this study are available from the corresponding author upon reasonable request.
